# MicroRNA as potential biomarker for severity, progression, and therapeutic monitoring in animal models of limb-girdle muscular dystrophy: a systematic review

**DOI:** 10.3389/fncel.2023.1233181

**Published:** 2023-12-07

**Authors:** Mayala Thayrine de Jesus Santos Oliveira, Talita Araújo Barbosa da Silva Santana, Marcela Câmara Machado Costa, Grasiely Faccin Borges, Felipe Silva de Miranda, José Slaibi-Filho, Wilson Barros Luiz, Luciene Cristina Gastalho Campos

**Affiliations:** ^1^Department of Health Sciences, State University of Santa Cruz, Ilhéus, Brazil; ^2^Laboratory of Applied Pathology and Genetics, State University of Santa Cruz, Ilhéus, Brazil; ^3^Escola Bahiana de Medicina e Saúde Pública, Salvador, Brazil; ^4^Public Policies and Social Technologies Center, Federal University of Southern Bahia, Itabuna, Brazil; ^5^Department of Biological Science, State University of Santa Cruz, Ilhéus, Brazil

**Keywords:** muscular dystrophies, neurodegenerative disease, biomarkers, microRNAs, animal model, neuropathology

## Abstract

Limb-girdle muscular dystrophies (LGMD) constitute a heterogeneous group of neuromuscular disorders in which there are alterations in proteins responsible for the preservation of muscle architecture and function, leading to proximal and progressive muscle weakness. There is, however, significant phenotypic and genotypic variation, as well as difficulty in establishing biomarkers that help to define pathogenic mechanisms and assess disease severity and progression. In this field, there is special attention to microRNAs, small non-coding RNA molecules related to the regulation of gene expression and, consequently, the production of proteins. Thus, this research aimed to verify the correlation between the expression of microRNAs and the severity, progression, and therapeutic response of LGMD animal models. A search was carried out in the PubMed, Embase, Scopus, ScienceDirect, Cochrane, and SciELO databases, with articles in English and without a time limit. The PRISMA 2020 checklist was used, and the protocol of this review was submitted to PROSPERO. The bibliographic survey of the 434 records found that 5 original articles met the inclusion criteria. The studies explored myomicroRNAs or miRNA panels with gene expression analysis. The analysis demonstrates that miR-1, 133a, and 206 are differentially expressed in serum and muscle. They change according to the degree of inflammation, fibrosis, muscle regeneration, and progression of the dystrophic process. MicroRNAs are up-regulated in dystrophic muscles, which are reversed after treatment in a dose-dependent manner. The present study inferred that miRs are essential in severity, progression, and therapeutic response in LGMD models and may be a useful biomarker in clinical research and prognosis. However, the practical application of these findings should be further explored.

## 1 Introduction

The hereditary information transmitted from progenitors to progeny supports the transgenerational perpetuation of characteristics. Nevertheless, numerous examples of hereditary phenotypic diversity elude the transference of traits exclusively through changes in the DNA sequence explained by Mendelian genetics (Skvortsova et al., 2018). Consequently, the term “epigenetic modifications” to heritable changes in gene expression that endure across cell divisions, regardless of alterations in the DNA nucleotide sequence. These modifications indicate a significant transformation in phenotype, while the genotype remains unaltered (Piletiè and Kunej, 2016; Morales et al., 2017).

Maintaining the stability of epigenetic information is imperative, given that alterations can lead to detrimental outcomes within the cell, encompassing aberrant gene expression and the induction of apoptosis. Epigenetic alterations assume a critical role in both the initiation and progression of numerous diseases, and they provide insights into complex disease characteristics like delayed onset, gender disparities, geographical influences, and variations in symptomatology. Accordingly, epigenetic mechanisms exert a momentous influence over the coordinated orchestration of processes such as replication, transcription, and repair. These mechanisms primarily encompass DNA methylation, modifications to histone proteins, and regulatory functions executed by non-coding RNAs, particularly microRNAs (Piletiè and Kunej, 2016; Morales et al., 2017).

MicroRNAs (miRNAs or miRs) are small molecules of non-coding single-stranded RNAs with a length of 18 to 25 nucleotides (Morales et al., 2017; Pös et al., 2018). These molecules play an essential role in gene expression modulation across diverse biological processes. They have a remarkable capacity to finely modulate up to 60% of protein-coding genes within the human genome, predominantly operating at the level of translation. These miRNAs participate in the orchestration of gene regulatory networks, and are integral to various physiological phenomena, spanning from cellular differentiation and proliferation to apoptosis and developmental processes. Notably, perturbations in their regulatory functions have been intricately linked to an array of disorders, including intricate conditions like muscular dystrophy (Catalanotto et al., 2016; Pös et al., 2018).

MicroRNAs (miRNAs) constitute an important source of non-invasive potential biomarkers due to their presence in a variety of human biofluids, including serum. Circulating MiRNAs (c-miRNAs) studies as biomarkers have been viable because of their abundance, stability within biofluid specimens, compatibility with cost-effective measurement techniques, and ease of exploring potential candidates when compared with analogous efforts aimed at discovering new biomarkers based on proteins. Beyond their analytical advantages, the utilization of c-miRNAs as biomarkers offers biological merits due to their specific expression patterns across tissues and their significant regulatory roles in both physiological and pathological processes (Coenen-Stass et al., 2017).

Thus, the dysregulated profile of miRNAs seems to be an interesting research avenue in neuromuscular diseases and the most recently investigated biomarkers are the expression of myomiRNAs (such as miR-1, miR-133a, miR-133b, and miR-206) as the measure of muscle miRNA expression can potentially inform about the pathophysiological processes that occur in dystrophic muscle (Coenen-Stass et al., 2017; Angelini et al., 2018).

Muscular dystrophies are a group of diseases of genetic origin that lead to the progressive loss of muscle tissue and that have histological characteristics of necrosis and muscle fiber regeneration, in addition to increased fibrosis and adipose tissue (Straub et al., 2018; Angelini, 2020). Given the diversity of molecules that form the muscle, different muscular dystrophies have been described, each possessing peculiar clinical, phenotypic, and genotypic characteristics. Among them, we can mention limb-girdle muscular dystrophy.

Limb-girdle muscular dystrophies (LGMD) are a heterogeneous group of neuromuscular diseases (NMD) characterized by predominantly proximal muscle weakness and caused by alterations in genes encoding proteins involved in the maintenance of muscle structure and activity (Narayanaswami et al., 2014; Straub et al., 2018). Mutations have been identified in more than 30 genes, associated with different subtypes, and presenting phenotypic and genotypic variability, which makes their diagnosis, prognosis, and treatment a real challenge (Nallamilli et al., 2018).

Although LGMD is classified as a rare condition with variable progression, the fact that some symptoms can be treated makes it essential to identify patients with a tendency to faster progression based on the identification of accurate biomarkers (Peric et al., 2018) that are capable of providing information about their correlation with clinical and progression characteristics (Thompson and Straub, 2016). It is known that the regulation of development, maintenance of homeostasis, and orchestration of metabolic pathways are widely acknowledged as essential, for the optimal operation of skeletal muscle. Consequently, a comprehension of the complex mechanisms underlying these processes assumes fundamental importance (Singh et al., 2020).

To address this challenge effectively, creating animal models is crucial for complex disorders like Limb-Girdle Muscular Dystrophy (LGMD) (Monani and De Vivo, 2014). These models offer valuable insights into the mechanistic details, structural features, and functional attributes of gene products. They also provide opportunities for therapeutic development. Systematically studying animal models across muscular dystrophies has consistently led to substantial progress in understanding and treating these conditions. (Ng et al., 2012). Thus, the use of microRNAs as potential biomarkers in LGMD using animal models can contribute to a better understanding of the pathophysiology and studies to assess severity and progression, and favor the development of therapies.

In this sense, the present study proposes to conduct a systematic review of the relationship between the expression of microRNAs and the severity, progression, and therapeutic monitoring in animal models of limb-girdle muscular dystrophy, to organize the published studies and analyze their results for a better understanding of the potential of microRNAs as a possible molecular tool in the clinical practice of limb-girdle muscular dystrophy.

## 2 Methods

This systematic literature review was conducted and reported according to the Preferred Reporting Items for Systematic Reviews and Meta-Analyses guidelines 2020 (PRISMA) statement (Page et al., 2021) and it was submitted to PROSPERO under ID 296358.

### 2.1 Search strategy

**FIGURE 1:**
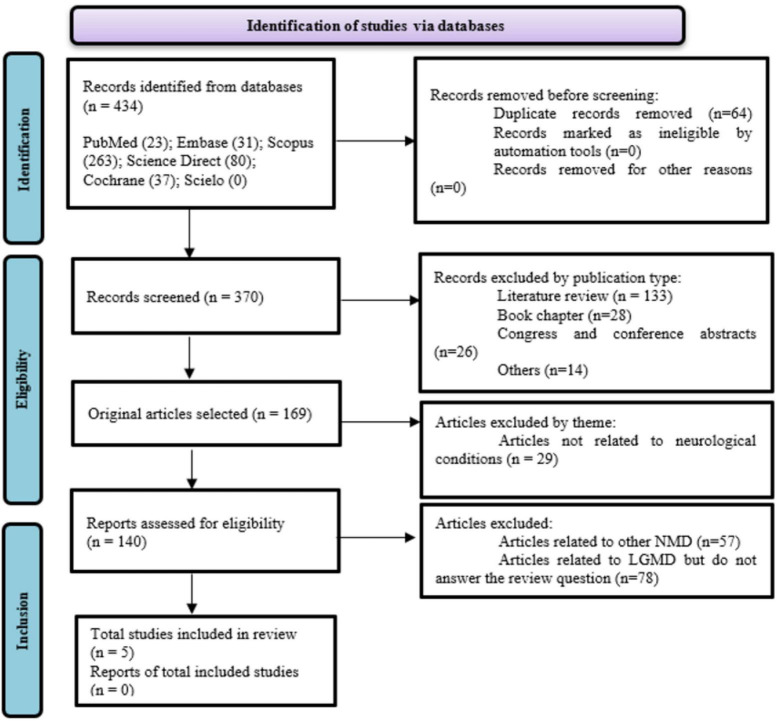
Flow diagram (PRISMA 2020).

We searched literature databases (Embase, PubMed, Scopus, ScienceDirect, Cochrane, and SciELO) to identify full-text articles reporting results from studies of microRNA expression in animal models of LGMD published through November 23, 2021 (Figure 1). The search query comprised four distinct sets of combinations, integrating Medical Subject Heading (MeSH) terms and topic field tags: 1# Muscular Dystrophies Limb-GirdleYalvac’s MeSH Terms] and microRNAYalvac’s MeSH Terms]; 2# “Muscular Dystrophies Limb-Girdle” or “Limb-Girdle Muscular Dystrophy” or “Sarcoglycanopathies” or “Limb-Girdle Syndrome” and “miRNA”; 3# “LGMD” and “miR”; 4# “Muscular Dystrophies Limb-Girdle” and “microRNA.”

### 2.2 Inclusion and exclusion criteria

For this review, only original articles that verified the expression of miRs in animal models of LGMD and the relationship with the severity and/or progression, and/or therapeutic monitoring of LGMD were analyzed. The other inclusion criteria were: (1) published in the English language; (2) have the study design defined (experimental studies—animal models); and (3) a description of results related to severity (assessment of muscle strength and/or respiratory impairment and/or muscle biopsy and/or MRI and/or use of other molecular biomarkers) and/or description of progression and/or therapeutic response; the method of measuring the described miR, regardless of the method used; analysis of miR expression measured in muscle tissue or blood (serum/plasma) or saliva samples.

The criteria for exclusion encompassed: (1) research involving duplicated databases; (2) assessments such as literature reviews, clinical case reports, meta-analyses, conference abstracts, letters to the editor, theses, dissertations, and book chapters; (3) investigations that solely stratified animal models with other muscular dystrophy subtypes; and (4) experimental studies lacking a control group or a comparative cohort.

### 2.3 Selection

Studies were selected in two stages. In the first step, two reviewers (MJ and TS) independently assessed the titles and abstracts of all identified references. Subsequently, a complete analysis of the chosen texts was conducted. Any disparities in the interpretation of articles during both phases were resolved through collaborative discussions and mutual agreement among the researchers. The third reviewer (LG) was available for cases where the two reviewers did not reach a consensus or when discrepancies arose.

### 2.4 Data collection

Reviewers collected the relevant information from all selected articles, including authorship, year of publication, country, study design, trials, results, and principal outcomes. After completion of the searches, a thorough check was carried out on the reference list of selected articles to certify that no publication was neglected. Reference management was done manually, and duplicate studies were removed.

### 2.5 Quality assessment of studies and risk of bias

It is considered essential to assess the quality of studies for an adequate understanding of them, therefore, the reviewers (MJ and TS) evaluated all selected articles according to the SYRCLE (Hooijmans et al., 2014) to judge the quality of evidence. If there is no consensus between the two authors, a third author (LG) was requested for a final decision.

## 3 Results

### 3.1 Study characteristics

The studies were published from 2013 to 2019, and they were carried out in two different countries: two in the United States (Yalvac et al., 2017; Verhaart et al., 2019); and three in France (Vignier et al., 2013; Israeli et al., 2016, 2019).

The selected studies evaluated the expression of miR in mice. One study performed the analysis in muscle (Yalvac et al., 2017), two in serum (Vignier et al., 2013; Verhaart et al., 2019), and two in muscle and serum (Israeli et al., 2016, 2019). Five subtypes of LGMD models were studied (LGMD R1, -R2, -R3, -R5, and -R6). The number of animals used in the experiment, by subtype, ranged from 1 to 18. A summary of the characteristics of the studies can be seen in Table 1.

**TABLE 1:** Characteristics of selected studies.

References	Subtype LGMD	Sample (*n*)	MiRs	Mir normalizer
Vignier et al., 2013	SGCA (LGMD R3)	6–8	517 miR	miR 29a, miR 30b
Vignier et al., 2013	SGCG (LGMD R5)	6–8	517 miR	miR 29a, miR 30b
Israeli et al., 2016	CAPN3 (LGMD R1) DYSF (LGMD R2) SGCA (LGMD R3) SGCG (LGMD R5)	1 each	miR 1, miR 133, miR 206 (Group 1) miR 149-5p, miR 193b, miR 378a- 3p (Group 2) miR 21, miR 31, miR 141-3p (Group 3)	miR 16 (serum) miR 93 (muscle)
Israeli et al., 2016	SGCA (LGMD R3)—Assess response to therapy.	5	miR 1, miR 133, miR 206 (Group 1) miR 149-5p, miR 193b, miR 378a- 3p (Group 2) miR 21, miR 31, miR 141-3p (Group 3)	miR 16 (serum) miR 93 (muscle)
Yalvac et al., 2017	CAPN3 (LGMD R1)	3–5 per group	miR 1, miR 206, miR 133a	U6snRNA
Israeli et al., 2019	SGCG (LGMD R5)—Assess response to therapy.	12	miR-1, miR-133a, mR133b, miR-206, miR-378a-3p, miR-30e, miR-149, and miR-193b	-
Verhaart et al., 2019	SGCA (LGMD R3)	18	cel-miR-39-3p, cel-miR-54-3p, cel-miR-238-3p, miR-1a-3p, miR-23a-3p, miR-30e-3p, miR-133a-3p, miR-133b-3p, miR-148a-3p, miR-206-3p, miR-434-3p, miR-451a-3p	cel-miR-39-3p, cel-miR- 54-3p and cel-miR-238-3p
Verhaart et al., 2019	SGCD (LGMD R6)	18	cel-miR-39-3p, cel-miR-54-3p, cel-miR-238-3p, miR-1a-3p, miR-23a-3p, miR-30e-3p, miR-133a-3p, miR-133b-3p, miR-148a-3p, miR-206-3p, miR-434-3p, miR-451a-3p	cel-miR-39-3p, cel-miR- 54-3p and cel-miR-238-3p

Subtitle: SGCA: α-sarcoglycanopathy (model); SGCG: γ-sarcoglycanopathy (model); SGCD: δ-sarcoglycanopathy (model); CAPN3: calpainopathy (model); DYSF: dysferlinopathy (model).

Regarding statistical analysis, all studies used a *p*-value of < 0.05 as statistically significant. In Vignier et al. (2013), differential expression was calculated using the 2^–ΔΔCt^ method, and miRNAs were considered differentially expressed beyond a threshold of a 1.5-fold change (FC). In Israeli et al. (2016), microRNAs expressions are expressed as a fold change (FC) relative to the control C57Bl/6 mouse. In Yalvac et al. (2017), summary statistics were reported as mean ± SEM. In Israeli et al. (2019), the relative expression was calculated using the 2^–ΔΔCt^ method. In Verhaart et al. (2019), values are presented as means ± standard deviation (SD), and histology, gene expression, miR levels, and heart—body weight ratio were analyzed with a two-way ANOVA between genotypes and age groups with Tukey’s multiple comparison test to correct for multiple testing.

Vignier et al.’s (2013) group performed a study with mice aged 9–11 weeks and compared the expression levels of sera miRNAs in pools of mice, collected from 6 to 8 mice per pool. First, a large-scale miRnome screen tested 517 mouse miRNAs. Of the 201 miRNAs detected, 37 were differentially expressed in at least one pathological strain, nine up-regulated and four down-regulated in SGCA while seven up-regulated and six down-regulated in SGCG. Second, miRNAs that were identified in the first screen, that is, the 37 differentially expressed and some additional differentially expressed miRNAs, as well as a collection of miRNAs that are known to be expressed in normal and pathological muscle, for a total of 87 miRNAs, being 10 up-regulated and 13 down-regulated in SGCA and 10 up-regulated and 12 down-regulated in SGCG (Vignier et al., 2013).

The data exhibited a stratification into three primary clusters: the dystrophin-associated protein complex (DAPC)-linked myopathies encompassing sarcoglycanopathy (SGC) and Duchenne muscular dystrophy (DMD), with their corresponding C57BL/6 control mice. The second and third clusters comprised other pathological mouse models, each paired with its respective control strain. The subdivision observed among the three primary clusters not only aligned with specific pathological presentations but also reflected the distinct genetic origins incorporated in the study. Moreover, the expression of 22 miRNAs was identified within these groupings (Vignier et al., 2013).

In the three models of DAPC-associated myopathy characterized by massive tissue destruction, the maximum value obtained from up-regulated miRNAs largely exceeded the maximum value obtained from down-regulated miRNAs. Strong activation of four miRNAs (miR-1, miR-133a, miR-133b, and miR-206) was identified in all three DAPC-associated myopathies. In addition to the activation of these myomiRNAs, dysregulation of various miRNAs in the serum was evident, many of them shared between the DAPC-associated myopathy models. Interestingly, the upregulation of miR-378, an additional myomiRNA, was observed along with elevated levels of miR-193b, miR-149, and miR-30a. Significantly, miR-122 demonstrated notable downregulation across all three instances of DAPC-associated myopathy. Furthermore, miR-31, known for its involvement in the regulation of dystrophin gene expression, was also consistently downregulated in contexts associated with DAPC-related pathologies (Vignier et al., 2013).

### 3.2 Severity and progression LGMD

#### 3.2.1 Analysis of serum miRNAs

In correlation, the serum creatine kinase (CK) levels were elevated in all three groups of degenerative myopathy that are linked with the DAPC. miR-200a was the only miRNA showing inconsistent dysregulation among all studied models. It exhibited downregulation in DAPC-associated pathologies while demonstrating upregulation in the two other pathological conditions. The underlying implications of serum miR-31 downregulation currently present challenges in terms of interpretation (Vignier et al., 2013).

Among the eight miRNAs exhibiting dysregulation in the Emery-Dreifuss muscular dystrophy (EDMD) model, three miRNAs, specifically miR-1, miR-133a, and miR-133b, displayed dysregulation in DAPC, although with reverse patterns. Consequently, the myofiber-enriched miRNAs exhibit upregulation in the serum of myopathies characterized by substantial muscle fiber destruction. On the other hand, myopathies linked to a more modest degenerative or regenerative process showed downregulation in the serum. This pattern concurred with the observed decrease in serum creatine kinase (CK) levels in individuals affected by EDMD. Additionally, pathologies primarily influencing skeletal muscle demonstrated a higher number of shared dysregulated circulating miRNAs compared to the MyBP-C-related hypertrophic cardiomyopathy model, which specifically targets cardiac tissues. It was further noted that no obvious advantage was seen in using miR-1, miR-133a, miR-133b, and miR-206, over CPK (Table 2).

**TABLE 2:** Summary of microRNA expression in animal models of LGMD.

Subtype	Tissue	miRs upregulated	miRs downregulated
CAPN3 (LGMD R1)	Serum	miR 1 (1 and 6 months) (Israeli et al., 2016) miR 133a (1 and 6 months) (Israeli et al., 2016) miR 206 (1 and 6 months) (Israeli et al., 2016)	
	Muscle	miR1 (12 weeks) (Yalvac et al., 2017) miR 206 (12 weeks) (Yalvac et al., 2017) (1 month) (Israeli et al., 2016) miR 31 (1 month) (Israeli et al., 2016) miR 21 (1 and 6 months) (Israeli et al., 2016) miR 142-3p (1 and 6 months) (Israeli et al., 2016)	miR-1 (4 weeks) (Yalvac et al., 2017) mir-133a (4 weeks) (Yalvac et al., 2017) miR-206 (4 weeks) (Yalvac et al., 2017)
DYSF (LGMD R2)	Serum	miR 1 (6 months) (Israeli et al., 2016) miR 133a (6 months) (Israeli et al., 2016) miR 206 (6 months) (Israeli et al., 2016)	
	Muscle	miR 206 (1 and 6 months) (Israeli et al., 2016) miR 31 (1 and 6 months) (Israeli et al., 2016) miR 21 (1 and 6 months) (Israeli et al., 2016) miR 142-3p (1 and 6 months) (Israeli et al., 2016)	
SGCA (LGMD R3)	Serum	miR 206 (Vignier et al., 2013) (1 and 6 months) (Israeli et al., 2016) miR133b (Vignier et al., 2013) miR 1 (Vignier et al., 2013) (1 and 6 months) (Israeli et al., 2016) miR 133a (Vignier et al., 2013) (1 and 6 months) (Israeli et al., 2016) miR 378 (Vignier et al., 2013) miR 193b (Vignier et al., 2013) miR 149 (Vignier et al., 2013) miR 30a (Vignier et al., 2013) miR 30d (Vignier et al., 2013) miR 709 (Vignier et al., 2013) miR 30e (Vignier et al., 2013)	miR 122 (Vignier et al., 2013) miR 672 (Vignier et al., 2013) (Vignier et al., 2013) miR 125a-5p (Vignier et al., 2013) miR 200a (Vignier et al., 2013) miR 199a-3p (Vignier et al., 2013) miR 195 (Vignier et al., 2013) miR 429 (Vignier et al., 2013) miR 151-3p (Vignier et al., 2013) miR 31 (Vignier et al., 2013) miR 26a (Vignier et al., 2013) miR 125b-5p (Vignier et al., 2013) miR 142-3p (Vignier et al., 2013) miR 152 (Vignier et al., 2013) mir 301b (Vignier et al., 2013) miR 93 (Vignier et al., 2013) mir 200b (Vignier et al., 2013)
	Muscle	miR 1 (Verhaart et al., 2019) miR 206 (Verhaart et al., 2019) (1 and 6 months) (Israeli et al., 2016) miR 31 (1 and 6 months) (Israeli et al., 2016) miR 21 (1 and 6 months) (Israeli et al., 2016) miR 142-3p (1 and 6 months) (Israeli et al., 2016)	miR 1 (6 months) (Israeli et al., 2016)
SGCG (LGMD R5)	Serum	miR 133b (Vignier et al., 2013) miR 133a (Vignier et al., 2013) (1 and 6 months) (Israeli et al., 2016) miR 206 (Vignier et al., 2013) (1 and 6 months) (Israeli et al., 2016) miR 1 (Vignier et al., 2013) (1 and 6 months) (Israeli et al., 2016) miR 378 (Vignier et al., 2013) miR 30d (Vignier et al., 2013) miR 193b (Vignier et al., 2013) miR 22 (Vignier et al., 2013) miR 149 (Vignier et al., 2013) miR 30a (Vignier et al., 2013) miR 106a(Vignier et al., 2013)	miR 125a-5p (Vignier et al., 2013) miR 31 (Vignier et al., 2013) miR 26b (Vignier et al., 2013) miR 142-3p (Vignier et al., 2013) miR 429 (Vignier et al., 2013) miR 26a (Vignier et al., 2013) miR 200a (Vignier et al., 2013) miR 122 (Vignier et al., 2013) miR 672 (Vignier et al., 2013) let 7g (Vignier et al., 2013) let 7l (Vignier et al., 2013) miR 215 (Vignier et al., 2013) miR 301b(Vignier et al., 2013)
	Muscle	miR 206 (1 and 6 months) (Israeli et al., 2016) miR 31 (1 and 6 months) (Israeli et al., 2016) miR 21 (1 and 6 months) (Israeli et al., 2016) miR 142-3p (1 and 6 months) (Israeli et al., 2016)	
SGCD (LGMD R6)	Muscle	miR 1 (Verhaart et al., 2019) miR 206 (Verhaart et al., 2019)	miR 133a (Verhaart et al., 2019) 133b (with the age)(Verhaart et al., 2019)
Common among models associated with DAPC	Serum	miR 206 (Vignier et al., 2013) miR 133b (Vignier et al., 2013) miR 133a (Vignier et al., 2013) miR 1 (Vignier et al., 2013) miR 378 (Vignier et al., 2013) miR193b (Vignier et al., 2013) miR 149 (Vignier et al., 2013) miR 30a (Vignier et al., 2013) miR 1, miR 133, miR 206 miR 149-5p, miR 193b, miR 378a- 3p miR 21, miR 141-3p	miR 301b (Vignier et al., 2013) miR 142-3p (Vignier et al., 2013) miR 31 (Vignier et al., 2013) miR 672 (Vignier et al., 2013) miR 200a (Vignier et al., 2013) miR 429 (Vignier et al., 2013) miR 122 (Vignier et al., 2013)

Verhaart et al. (2019) conducted a study that assessed grip strength and limb suspension tests for all limbs in male wild-type mice Sgca^–/–^, Sgcd^–/–^, and C57BL/6J (*n* = 18 per genotype), including two- and four-limb suspension tests on consecutive days. These assessments were carried out twice a month in 4–8, 16, and 24-week-old mice. At each time point, six male mice per genotype were sacrificed via cervical dislocation and dissected for muscle analysis, while the remaining mice continued the functional testing protocol. Additionally, an assessment of male Sgca^–/–^ and Sgcd^–/–^ mice’s *in vivo* muscle function was conducted twice-weekly over intervals of 4, 12, or 20 weeks and compared to wild-type C57BL/6J mice of like body mass and age. The four-limb grip strength test revealed no significant differences in muscle strength between the genotypes. To assess muscle function and susceptibility to fatigue, both two and four-limb suspension tests were performed. In both evaluations, wild-type mice performed better than Sgca^–/–^ and Sgcd^–/–^ mice (*p* < 0.01). Muscle function declined with age, and there were no differences between Sgca^–/–^ and Sgcd^–/–^ mice (Verhaart et al., 2019).

A thorough examination of respiratory functions using comprehensive full-body plethysmography showed decreased respiratory range relative to body weight in Sgca^–/–^ and/or Sgcd^–/–^ mice compared to wild-type mice, especially at 8 and 16 weeks. Additionally, the respiratory rate decreased consistently in both LGMD models at all ages, with significant differences in Sgca^–/–^ and Sgcd^–/–^ mice at 8 and 16 weeks. However, despite age-related variations, respiratory function was stable across all genotypes (Verhaart et al., 2019).

Muscle tissue of the gastrocnemius, quadriceps, triceps, and diaphragm were examined using H&E staining to detect damage such as necrosis, fibrosis, inflammation, and regenerated fibers. Both Sgca^–/–^ and Sgcd^–/–^ mice had lower muscle quality vs. wild-type mice at 8 weeks old. The diaphragmatic muscle exhibited more severe degenerative alterations compared to the musculature of the limbs. There was no significant difference in muscle damage between pathological models, and the damage remained stable as the mice aged with the diaphragm being the most affected (Verhaart et al., 2019).

Sirius red staining was performed on muscle sections to evaluate the extent of fibrosis. Muscles in Sgca^–/–^ and Sgcd^–/–^ models had high levels of fibrosis from 8 weeks of age, with the diaphragm being the most affected, and the fibrotic lesions increased in some muscles with age. In addition, genes related to fibrosis–Col1a1, Lox, Pdgfrα, Ctgf, and Ltbp4–showed interesting trends in mice with LGMD. Sgca^–/–^ and Sgcd^–/–^ mice showed significant upregulation of Col1a1, Lox, and Pdgfrα from an early stage, with a slight decrease in Col1a1 levels in the gastrocnemius and triceps muscles as the mice aged. The levels of Pdgfrα were found to be higher in all muscles, except for the diaphragm. The expression of Ctgf and Ltbp4 showed moderate changes, with an increase in Ctgf expression observed in Sgcd^–/–^ males but not in Sgca^–/–^ males, and an increase in Ltbp4 expression observed only at 24 months, primarily in Sgcd^–/–^ males (Verhaart et al., 2019).

To determine the infiltration of adipose tissue, the Pparγ expression was assessed, a key regulator in adipocyte differentiation. Thus, Pparγ expression was upregulated in both Sgca^–/–^ and Sgcd^–/–^ mice, with the gastrocnemius muscle being the most affected. However, there was no significant difference in Pparγ expression between the wild-type and LGMD mouse strains in the diaphragm. Laminin stain was used for a thorough analysis of the distribution of fiber sizes in multiple muscles as accurately to distinguish individual muscle fibers. Interestingly, a trend toward smaller sizes in both LGMD models was found indicating regenerative processes. Diaphragm muscles had smaller fiber sizes overall, even in wild-type mice. Importantly, they didn’t detect significant changes in muscle fiber sizes over time. Additionally, there were no significant differences in fiber sizes between the two LGMD models (Verhaart et al., 2019).

An analysis of the expression profiles of various markers linked with regenerative processes, such as Myh3, MyoG, Nox2, and Stat3, showed that the majority of these markers were upregulated in LGMD models from the age of 8 weeks. Moreover, there were no significant changes over time. Notably, Myh3, which is primarily expressed in newly regenerated fibers, was scarcely present in wild-type mice. In Sgca^–/–^ and Sgcd^–/–^ mice, however, significantly higher levels were found in all muscles analyzed at 8 weeks, in the triceps and diaphragm at 16 weeks, and in the diaphragm at 24 weeks. The LGMD mice had early regeneration, indicated by an increase in MyoG. The increase of Nox2 showed inflammation and regeneration processes in all muscle types of Sgca^–/–^ and Sgcd^–/–^ mice, regardless of age. Stat3 showed a significant increase in the gastrocnemius and quadriceps of Sgca^–/–^ and/or Sgcd^–/–^ mice at 24 weeks of age and in the triceps muscle of both dystrophic models at all-time points, except for Sgca^–/–^ mice at 8-week-old. There was a small increase over time in the quadriceps of Sgca^–/–^ mice (Verhaart et al., 2019).

Dystrophic muscle is characterized by the infiltration of immune cells. To quantify the magnitude of the inflammatory response, the expression levels of CD68 and LGALS3 were evaluated. These markers exhibited increased expression across various muscle groups in Sgca^–/–^ and Sgcd^–/–^ mice at most time points. Similarly, to the observations related to fibrosis and regeneration, age-related distinctions were not evident. To probe the potential involvement of calcification in the pathogenic processes within Sgca^–/–^ and Sgcd^–/–^ mice, muscle sections were subjected to staining with Alizarin Red. While calcified fibers were notably absent in wild-type mice, both murine models of sarcoglycanopathy exhibited clusters of calcified fibers within their skeletal muscles, regardless of the animals’ chronological ages. It’s worth highlighting in order to probe the potential involvement of calcification in the pathogenic processes within Sgca^–/–^ and Sgcd^–/–^ mice, muscle sections were subjected to staining with Alizarin Red. While calcified fibers were notably absent in wild-type mice, both murine models of sarcoglycanopathy exhibited clusters of calcified fibers within their skeletal muscles, regardless of the animals’ chronological ages. It’s worth highlighting that the extent of calcification demonstrated variability among individual specimens (Verhaart et al., 2019).

With a limited sample of 24-week-old mice, serum from Sgca^–/–^, Sgcd^–/–^, and 34-week-old C57BL/6J males, collected in a study by the authors of previous natural history, was used. Elevated levels of miR-1a and miR-206, both known to instigate myogenic differentiation and abundant in muscle tissue, were predominantly detected in young Sgca^–/–^ and Sgcd^–/–^ mice. On the other hand, no significant distinctions were observed for miR-30e and miR-148a. The active myoblast proliferation in Sgca^–/–^ and Sgcd^–/–^ males was indicated by up-regulated of miR-133a and miR-133b. Across all genotypes, a small reduction in expression during aging was observed, with statistical significance achieved solely in Sgcd^–/–^ mice. Additionally, the downregulation of miR-434, which functions to inhibit apoptosis was decreased with aging. However, no differences emerged between the LGMD models and wild-type mice (Verhaart et al., 2019).

#### 3.2.2 Analysis of muscle microRNAs

In Yalvac et al.’s (2017) group conducted a study using animal models with a deficiency in calpain. The researchers induced muscle necrosis and regeneration in the gastrocnemius muscles of CAPN3-KO mice through multiple cycles of CTX injections. After 4 and 12 weeks of the last CTX induction, the fiber types observed were slow-twitch oxidative (STO), fast-twitch glycolytic (FTG), and intermediate fast-twitch oxidative (FTO). Additionally, fibers that displayed a lobulated pattern of structural change were identified (Yalvac et al., 2017).

The muscle tissue of CAPN3-KO mice had significantly more STO/type 1 small fibers and a smaller average diameter of muscle fibers compared to the healthy control at both time points. This decrease in diameter was mainly due to the STO fibers having a minimal expansion in their diameter after the 12-week interval following the last CTX induction, compared to the 4-week interval. Additionally, the radial growth of fast-twitch fibers with glycolytic/oxidative properties appeared to be impacted less than that of the STO fibers. However, this reduced growth was still significantly better than the STO fibers and remained better compared to the controls (Yalvac et al., 2017).

Moreover, there was a markedly elevated STO:FTG/O ratio in the CAPN3-KO group when contrasted with the control cohort. This signifies that in muscles afflicted with CAPN3 deficiency, following multiple rounds of necrosis and subsequent regeneration, there is a prevalence of smaller fibers endowed with oxidative potential. Notably, the CAPN3-KO mice exhibited diminished muscle mass, and the intrinsic diameter of muscle fibers within the non-regenerating gastrocnemius muscle on the contralateral side was notably smaller than in the controls. This size reduction was observable across both fast-twitch and slow-twitch fibers. Furthermore, the baseline muscle fiber composition of the CAPN3-KO mice exhibited an increased prevalence of fibers, demonstrating higher oxidative capacity when compared to the wild-type group (Yalvac et al., 2017).

In muscles without CAPN3 protein, the amount of connective tissue after regeneration increased significantly at 4 and 12 weeks compared to the control group. At the 12-week mark, there was a trend toward more fibrosis than 4 weeks, though it didn’t reach statistical significance. As expected, healthy muscles had less connective tissue and a larger fiber diameter. These observations are similar to the characteristics of LGMDR1, which include small type 1 fibers with a lobulated structure and increased endomysial and perimysial connective tissue (Yalvac et al., 2017).

MicroRNA expression profiles were evaluated after repeated cycles of necrosis/regeneration events in CAPN3-KO muscles and controls. No statistically significant differences in myomiR expression profiles were found within the uninjected contralateral muscles between CAPN3-deficient and control groups at the 4- and 12-week time points. Therefore, the data from regenerating muscles were normalized against the levels obtained from non-injected muscles in the 4-week post-injection cohorts, which served as a baseline within each group. Notably, an increase of 22-fold was described in miR-206 transcript levels in the CTX-treated control muscle compared to baseline levels at the 4-week time point, which subsequently decreased to 3.6-fold at the 12-week time point after the last CTX induction (Yalvac et al., 2017).

In muscles undergoing the regenerative process in the absence of the CAPN3 gene, expression levels of miR-206 were up-regulated, with a substantial 15-fold increase compared to the baseline at the 4-week mark. Subsequently attenuating to a 10-fold elevation relative to the baseline at the 12-week time point. This scenario highlights a significant reduction in miR-206 expression over time compared to the control, as observed 12 weeks after injection. Similarly, alterations in the expression levels of miR-1 and miR-133a also manifested a lesser magnitude of change in comparison to their wild-type counterparts at both time points, following a parallel pattern of more gradual decline (Yalvac et al., 2017).

#### 3.2.3 Analysis of muscle and serum miRNAs

In the study developed by Israeli et al. (2016), the usefulness of circulating miRNAs for therapeutic monitoring was investigated. In the first part of the study, the modified expression of these miRNAs in two classifications of muscular dystrophy was examined, the initial one retaining an intact expression of the dystrophin-linked glycoprotein complex (DAPC) (DMD, SGCA, and SGCG) and second category involving the compromised structural integrity of the DAPC (CAPN3, DYSF, healthy controls). Three categories of microRNAs were analyzed: distromiRs (miR-1, miR-133, and miR-206); the modified expression of miRNAs in serum of 3 MD mouse models (miR-149-5p, miR-193b, and miR-378a-3p), and dysregulated miRNAs in MD mouse muscle biopsies associated with previous damage such as fibrosis, myoblast proliferation and immune cells infiltration (miR-21, miR-31, and miR-142-3p) (Israeli et al., 2016).

Thus, in 2016 Israeli’s group, compared five mouse models with healthy C57Bl/6 controls at two time points (1 and 6 months). KO-Sgca mice were used as models for LGMD R3 (α-sarcoglycanopathy), KO-Sgcg mice for LGMD R5 (γ-sarcoglycanopathy), and mdx4cv mice for DMD (all considered high regeneration models—HRM). The KO-Dysferlin mouse was used as a model for LGMD R2, and the KO-Capn3 was used as a model for LGMDR1, from mutations in the Dysf gene and the Capn-3 genes, respectively. Within the context of LGMDR1, the integrity of expression and subcellular localization of the dystrophin-associated glycoprotein complex (DAPC) remain preserved, distinguishing them as low-regeneration models (LRM). First, the expression levels of miRs, miR-1, miR-133a, and miR-206 were quantified in the serum of the five models. The miRs were up-regulated in the serum of all disease models at 1 and 6 months (except 1-month KO-Dysf). Serum distromiR upregulation was generally greater in HRM than in LRM (except for KO-Sgca at 6 months of age), and greater in 6-month-old mice than in 1-month-old mice (Israeli et al., 2016).

The analysis of microRNA expression was expanded to include the gastrocnemius (Ga) muscle in the same models and age groups. With the knowledge that miR-21, miR-31, and miR-142-3p are to be involved in three key processes that characterize muscular dystrophy, the expression of these three specific miRNAs was measured in addition to the distromiRs. The miR-31 and miR-21 expression levels were increased in dystrophic muscle, suggesting that miR-31 may be involved in regenerating myoblast formation and miR-21 may be involved in the cellular fibrotic cascade. Overall, muscle miRNA dysregulation, comprising up and downregulation, was greater at 6 months than at 1 month (Israeli et al., 2016).

Muscle biopsies show decreased miR-1 and miR-133a levels in high regeneration models, with only miR-1 showing statistical significance in KO-Sgca and mdx models at 6 months of age. MiR-206 and miR-31 were elevated in most high regeneration models, while miR-21 and miR-142-3p showed minimal upregulation in low regeneration models but were higher in high regeneration models due to infiltrating inflammatory cells. The increased expression of miR-142-3p is mainly due to CD45-positive mononuclear cells in HRM KO-Sgca muscle. Thus, data support that dystromiR dysregulation in MD occurs, at least partially, independently of pathophysiological mechanisms related to DAPC. Furthermore, an inverse dysregulation of miR-1 and miR-133a was observed in the context of muscular dystrophy, exhibiting a contrasting pattern between serum and muscle compartments. Specifically, these microRNAs displayed an elevation in serum levels while concurrently exhibiting a reduction in muscle tissue (Israeli et al., 2016).

#### 3.2.4 Monitoring therapeutic

The evaluation of the biomarker for therapeutic monitoring requires the investigation of the correlation between its expression or activity and the therapeutic benefit (Table 3). In the second part of the Israeli study (2016), the possibility of using circulating miRNAs as surrogate biomarkers in a mouse model of gene therapy was investigated. In this part of the investigation, the KO-Sgca mice model (LGMD R3) was used to assess miRNAs as potential biomarkers for monitoring in a preclinical gene transfer study. Specifically, a recombinant adeno-associated virus (rAAV9) carrying human α-sarcoglycan cDNA (SGCA) controlled by the desmin promoter was administered via intravenous injection to juvenile KO-Sgca mice at 5 weeks of age (*n* = 5). The mice were treated with escalating doses of the recombinant vector, including 4 × 10^12, 2 × 10^13, and 4 × 10^13 viral genome copies (vg/kg). Sgca–null (α-sarcoglycan deficient mouse) and C57Bl/6 (healthy mouse) injected with PBS (saline) were used as negative and positive controls, respectively (Israeli et al., 2016).

**TABLE 3:** Summary of the studies about miRs and response therapeutic in animal models LGMD.

Subtype	Therapy	Sample	Upregu-lated	Down-regulated
SGCA-KO (Israeli et al., 2016)	rAAV2/8-desm-hSGCA	Serum		miR 1 miR 133 miR 206 miR 149-5p miR 193b miR 378a- 3p miR 21 miR 141-3p
		Muscle	* miR 1 miR 133 miR 149-5p miR 193b miR 378a- 3p	miR 206 miR 21 miR 31 miR 141-3p
SGCG-KO (Israeli et al., 2019)	AAV8-desm-hSGCG	Serum	* miR-1 miR-133a mR133b miR-206 miR-378a-3p miR-30e miR-149 miR-193b	** miR-1 miR-133a mR133b miR-206 miR-378a-3p miR-30e miR-149 miR-193b

Subtitle: *D0 concerning the healthy control and after the escape, test to the untreated dystrophic group.

**After treatment and dose-dependent.

Expression and spatial distribution of the transgenes were evaluated by the following techniques: RT-qPCR, immunohistochemistry, and Western blot analysis. These analyses demonstrated an increment in both mRNA and protein expression that corresponded directly with the administered dosage of the recombinant vector. Positive outcomes were evident in the treated subjects, manifesting as enhancements in overall tissue structure and a decline in fibrosis. Additionally, the presence of inflammatory infiltration was evaluated using Sirius Red and CD11b staining, revealing a notable reduction. The extent of this response was quantitatively examined through the expression of a specific set of marker genes associated with muscular dystrophy by RT-qPCR (Israeli et al., 2016).

Furthermore, Israeli et al. evaluated the expression levels of various markers in MD48, including Myh8 (myosin heavy chain 8), which is important in muscle recovery, CD11b a marker of muscle inflammation, and the Col6a (an isoform of collagen 6), a biomarker associated with fibrotic tissue. These markers were significantly upregulated in untreated KO-Sgca mice but gradually decreased following treatment with varying viral doses. Additionally, the “escape” test, involving the measurement of mouse-generated escape force, also showed an improvement in muscle strength in treated KO-Sgca mice. The level of improvement was dose-dependent and eventually reached a comparable level to that of healthy controls for intermediate and high doses. Taken together, these results suggest that gene transfer treatment was effective in restoring muscle architecture and function in mice (Israeli et al., 2016).

The expression of several distromiRs including miR-1, miR-133a, miR-206, miR-378a-3p, and the miRNAs miR-149-5p, and miR-193b-3p was quantified in treated mice serum. The serum expression levels were evaluated at three distinct moments, firstly before injection (D0), followed by assessments at 14 and 56 days after gene delivery. At D0, KO-Sgca mice showed higher expression levels of all miRNA than healthy mice. However, in KO-Sgca mice treated, miRNA levels were downregulated in a dose-dependent manner. By day 14, except for miR-149-5p, all the other miRNA expression levels were not significantly different compared to healthy mice injected with PBS, particularly in the higher dose-treated groups (Israeli et al., 2016).

Furthermore, in the high-dose treated group, on day 14, the expressions of all studied miRNAs were significantly different from the untreated dystrophic group (KO-PBS). Similar results were obtained 56 days after transduction, except that the intermediate dose results were closer to the high dose. Taken together, the data show that the dysregulation of the serum miRNA profile was reduced in mice treated for all tested miRNAs, in direct relation to the increase in recombinant vector doses, transgene expression, and recovery of muscle function. MiRNA expression in muscle biopsies was assessed at the study’s conclusion. This analysis involved the dystromiRs class, which includes the classic miR-1, miR-133, and miR-206, alongside the recently introduced miR-149-5p, miR-193b-3p, and miR-378a-3p. Additionally, the study included three miRNAs known to be dysregulated in MD: miR-21, miR-31, and miR-142-3p. Notably, the expression of miR-1 and miR-133a, previously identified as down-regulated intracellularly in the muscles of 1- and 6-month-old KO-Sgca mice exhibited the expected down-regulation in muscle at the initial time point (D0) (Israeli et al., 2016).

Likewise, levels of miR-378a-3p, miR-193b-3p, and miR-149-5p were down-regulated in gastrocnemius muscle in MD mice. The levels of these miRNAs were elevated in the muscles of treated mice, demonstrating an association with increasing amounts of the therapeutic viral vector. Conversely, miR-206, miR-31, miR-21, and miR-142-3p, which were initially overexpressed in untreated dystrophic mice, displayed a decrease in expression corresponding to the viral doses administered. The expressions of all miRNAs significantly differed in at least one of the two higher-dose vector groups when compared to untreated dystrophic mice. Furthermore, at the highest vector dose, the expressions of five out of the nine examined miRNAs were no longer significantly dysregulated in comparison to healthy control mice. Collectively, the data suggest that 90 days after transduction, the muscle profiles of all assessed miRNAs exhibited trends toward normalization in the treated mice. This trend correlated with the viral dose administered and the restoration of muscle function (Israeli et al., 2016).

In Israeli et al. (2019) published a study like the second step of the one described above, but to assess the potential of gene transfer to correct SGCG deficiency, using a rAAV2/8 vector carrying the human SGCG cDNA under the transcriptional control of the desmin promoter. Viral production was validated by intramuscular injection into the tibialis anterior (TA) of Sgcg^–/–^ mice (*n* = 3 animals per group) with a dose of 1 × 10^10^ viral genome/TA. Subsequently, 1 month passed, a complete assessment of the muscles was conducted, and the expression levels of SGCG were verified through the utilization of immunohistochemical analysis applied to cross-sectional samples (Israeli et al., 2019).

Although the muscles Sgcg^–/–^ did not show any visible staining, the ones that were treated with AAV had staining in almost all of their myofibers. The staining was correctly located in the protein membrane, demonstrating a decrease in dystrophic features when examined histologically. These results confirmed the effectiveness of AAV8-desm-hSGCG. Later on, a study was conducted to examine the reactions of male and female animals to intravenous administration of the vector through the tail vein when they were a month old. The study involved three different doses: 4.5 × 10^12^ vg/kg, 1.5 × 10^13^ vg/kg, and 4.5 × 10^13^ vg/kg to evaluate the gene therapy dose-response (Israeli et al., 2019).

Various limb muscles (gastrocnemius, deltoid, heart, gluteus, extensor digitorum diaphragm, soleus, TA, triceps crural, quadriceps, psoas and longus), as well as organs (lungs, kidneys, spleen, liver), were evaluated 1 month after the injection (*n* = 10 per group). In most animals that received the lowest dose of vector, the proportion of ɣ-sarcoglycan positive fibers was estimated to be less than 5%. In the group of animals injected with the intermediate dose, 25–75% of the analyzed muscle fibers expressed the ɣ-sarcoglycan protein. Therefore, in mice that received the highest dose of the vector, 75–100% of the myofibers expressed the protein (Israeli et al., 2019).

Histological analysis also illustrated the dose-effect of vector administration. Quantitative assessment of transgene expression in the psoas muscle through Western blot analysis confirmed the expected dose-response. A semiquantitative analysis of ɣ-sarcoglycan expression in the total cross-section of different muscles (left and right sides of mice) was evaluated using an image-based high-throughput method. Immunostaining of serial sections with α- and β-sarcoglycans was also performed and showed a perfect correlation with the presence of proteins in different fibers, indicating the reconstitution of the sarcoglycan complex on the sarcolemma (Israeli et al., 2019).

Different levels of fiber degeneration were observed in various striated muscles of mice who received varying vector dosages. Mice who received intermediate doses showed mild to moderate dystrophic alterations in most muscles, while those who received the highest dose showed minimal fiber degeneration. Additionally, no discernible lesions were found in heart histological sections across all animal groups. A significant correlation was found between the proportion of centronuclear myofibers and vector concentrations. Higher doses were linked to lower scores of centrally nucleated myofibers (Israeli et al., 2019).

Finally, all of the mice underwent an escape test after the functional analysis. The results demonstrated a gradual correction of strength deficits in both male and female subjects, achieving a level similar to wild-type mice only with the highest dose administration. Furthermore, as an initial step in safety evaluation, the organs (liver, spleen, lungs, and kidneys) of mice previously treated with a high vector dose were subjected to histological analysis. These observations revealed that animals injected with high vector doses exhibited no notable organ lesions, indicating a favorable safety profile (Israeli et al., 2019).

An additional experiment was conducted to determine the effects of vector injection over a longer time and on physical performance. Ten female mice were administered with three doses as described in the previous experiment and were sacrificed 3 months after injection. Before sacrifice, the mice underwent an escape test. Blood was collected at three different times: at the start of the experiment, 1 week before sacrifice, and after the escape test. The results of the histological and functional analyses confirmed the dose effect of vector injection observed in the earlier experiment. Serum CK and distromiRs (miR-1, miR-133a, miR-133b, miR-149, miR-30e, miR-206, miR-378a-3p, and miR-193b) levels were evaluated. As per previous studies, the serum expression levels of all the studied miRNAs were higher in the KO-Sgcg mice than in the healthy control mice at the D0 time point, before injection (Israeli et al., 2019).

In KO-Sgcg treated mice, serum miRNAs, as well as CPK levels, were dose-dependently down-regulated before the escape test. However, after the escape test, a substantially increased level of miRNAs and CPK was observed compared to the untreated dystrophic group. Together, the data show that plasma miRNA profile dysregulation was reduced in mice treated for all tested miRNAs, in direct relation to increased recombinant vector dose and with functional muscle recovery, where there is no mechanical stress involved. In contrast, under conditions of mechanical stress, a decrease in miRNA levels was exclusively observed in mice administered the highest dosage. This phenomenon implies that optimal muscle recuperation is imperative for supporting resistance against mechanical stress (Israeli et al., 2019).

#### 3.2.5 Assessment of study quality and risk of bias

In summary, the following items are adopted in the SYRCLE scale by Hooijmans et al. (2014): (1) Was the allocation sequence adequately generated and applied?; (2) Were the groups similar at baseline or were they adjusted for confounders in the analysis?; (3) Was the allocation to the different groups adequately concealed; (4) Were the animals randomly housed during the experiment?; (5) Were the caregivers and/or investigators blinded from knowledge of which intervention each animal received during the experiment?; (6) Were animals selected at random for outcome assessment?; (7) Was the outcome assessor-blinded?; (8) Were incomplete outcome data adequately addressed?; (9) Are reports of the study free of selective outcome reporting?; and (10) Was the study free of other problems that could result in high risk of bias? (2014, Table 2, p. 45; Hooijmans et al., 2014).

The studies were uniform in items 1 and 8 to 10, with differences, and with uncertain or negative results in items 3–7. It should be noted that there is no score and classification for this scale. A breakdown of the notes for each study can be found in Table 4.

**TABLE 4:** Assessment of study quality of selected studies.

References	1	2	3	4	5	6	7	8	9	10
Vignier et al., 2013	No	Unclear	No	No	No	No	No	Yes	Yes	Yes
Yalvac et al., 2017	No	Yes	No	No	No	No	No	Yes	Yes	Yes
Israeli et al., 2016	No	Yes	Unclear	No	Unclear	Unclear	Unclear	Yes	Yes	Yes
Israeli et al., 2019	No	Yes	No	No	No	No	No	Yes	Yes	Yes
Verhaart et al., 2019	No	Yes	No	Unclear	Unclear	Unclear	Unclear	Yes	Yes	Yes

## 4 Discussion

Muscular dystrophies are a group of inherited diseases that primarily affect muscle tissue. Its symptoms commonly compromise the quality of life, and thus muscular dystrophies need reliable and measurable biomarkers that will monitor disease severity and progress as well as evaluate potential therapeutic approaches (Koutsoulidou and Phylactou, 2020).

Altered expression of microRNAs is involved in skeletal muscle homeostasis in health and disease. MyomiRs promote muscle cell differentiation and development by targeting repressors of skeletal muscle cell differentiation (Singh et al., 2020). MiR-1 promotes myogenesis by targeting histone deacetylase 4 (HDAC4) and a transcriptional repressor of the muscle-specific transcription factor, MEF2; thereby, promoting the expression of MEF2, which increases the expression of miR-1 and accelerating the differentiation of muscle fibers (Chen et al., 2006, 2010). It is postulated that miR-206 indirectly down-regulates Id1-3 and MyoR, myogenic transcription inhibitors factors like MyoD. This positive feedback loop between the myogenic transcription factors and muscle-specific miRNAs should push the balance toward differentiation, and it can be very significant in keeping muscle cells in a permanently differentiated state (Hak et al., 2006).

In the study developed by Vignier et al. (2013) and the first stage of the study by Israeli et al. (2016), it was possible to observe an association of miRNA dysregulation concerning severity, in a grouped way, between the models of high and low regeneration. The severity of Duchenne muscular dystrophy (DMD) might be modulated by different factors, including miRNAs. It was demonstrated that dystrophic mice additionally globally lacking miR-378 Yalvac’s double-KO (dKO) animals] exhibited better physical performance and improved absolute muscle force compared with mdx mice. Accordingly, markers of muscle damage in serum were significantly decreased in dKO mice, accompanied by diminished inflammation, fibrosis, and a reduced abundance of regenerating fibers within muscles. The lack of miR-378 also normalized the aggravated fusion of dystrophin-deficient muscle satellite cells. RNA sequencing of the gastrocnemius muscle transcriptome revealed fibroblast growth factor 1 (Fgf1) as one of the most significantly downregulated genes in mice devoid of miR-378, indicating FGF1 as one of the mediators of changes driven by the lack of miR-378 (Podkalicka et al., 2020).

Other miRs reported in selected studies have already been reported in association with cardiac alterations, cardiomyocyte development, and muscle fiber differentiation and transformation (miR30 family, mir-31 and miR-709) (Zaharieva et al., 2013; Stepicheva and Song, 2016; Zhang et al., 2016; Su B. et al., 2020; Su Y. et al., 2020; Huang et al., 2021). While miR-149-5p, miR-141-3p, and miR-193b have been associated with vascular smooth muscle cell proliferation, invasion, and migration (Zhang et al., 2019, 2020; Ren et al., 2021).

The finding of dysregulation, however, should not necessarily be interpreted as a causal link. Despite being related to fibrosis (Zanotti et al., 2015), the absence of miR -21, for example, when studied in congenital muscular dystrophy with laminin deficiency (LAMA2-CMD), which is not one of the LGMD subtypes, did not change the degree of dystrophy, indicating that it may not be involved in the development of fibrosis in this type of condition (Moreira Soares Oliveira et al., 2017).

There is an intersection between severity, progression, and difficulty in establishing well-defined criteria that allow us to assess how and why miRNA dysregulation occurs. Thus, from studies by the groups of Israeli et al. (2016), Yalvac et al. (2017), and Verhaart et al. (2019), data can be extracted that contributes to the understanding of the effect of miRNAs on the progression of limb-girdle muscular dystrophy.

The results by Verhaart showed that especially myomiRs involved in myoblast proliferation and differentiation (e.g., by affecting MyoG and/or MyoD signaling), such as miR-1a, miR-133a/b, and miR206 (Eisenberg et al., 2009; Koutsoulidou and Phylactou, 2020) were increased in the bloodstream of Sgca^–/–^ and Sgcd^–/–^ mice at most ages, suggestive of ongoing regeneration and damage. This confirms the results in another study in this review showing an increase of these miRNAs in Sgca^–/–^ mice (Vignier et al., 2013).

On the other hand, aberrant regeneration resulting from impaired myotube fusion after segmental necrosis has been proposed as the main cause of fibrosis. The results by Yalvac showed this it should be noted that the miR-206 upregulation level seen before the very late stage of calpainopathy is modest compared to robust regeneration examples like those seen in the WT muscle after CTX-induced injury, where miR-206 dramatically increases in regeneration muscle fibers (Yuasa et al., 2008).

Collectively, these findings show similar patterns of microRNA dysregulation in the muscle of LGMD animal models. Especially, along with the downregulation of miR-206, up-regulation of TGF-β underlines its important regulatory role in the stress signal mediation process, leading to a phenotypic change (Mendell and Olson, 2012).

The development of gene therapies for neurogenetic conditions, such as LGMD, is a palpable reality. Understanding, however, how to non-invasively assess response to therapy is a challenge that cannot be overlooked. The reduction of myomiR expression levels after treatments was observed in previous studies using exon-skipping and morpholino oligomer-mediated dystrophin restoration therapies in DMD mice (Cacchiarelli et al., 2011; Roberts et al., 2012, 2013). These results show a promising potential for the use of these miRNAs as pharmacodynamic biomarkers in DMD.

However changes in myomiRs are not restricted to muscular dystrophies. A reduction in muscle-specific miRNAs has been reported under nusinersen treatment in pediatric patients with SMA types II and III. Although more studies are needed, the overall findings highlighted the relevance of miR-133a, -133b, -206, and -1 in pathogenicity processes underlying neuromuscular disorders and supported their potential as non-invasive biomarkers to monitor disease progression and measure the effectiveness of therapeutic interventions (Bonanno et al., 2020). The usefulness of using microRNAs to monitor therapy can even go beyond gene therapies. In another study, Pegoraro et al. showed a significant decrease in levels of miR-133a, -133b, -206, and -1, after physical training in ALS patients (Pegoraro et al., 2019).

In this review, two studies conducted by the same group, evaluated the therapeutic response in animal models with LGMD to gene therapy, associated with the expression of miRNAs. Even though they are different subtypes, in both studies it was possible to observe a pattern in the expression of miRNA.

In the first study, there was dysregulation of all tested miRNAs, both up and down in serum and muscle, which was reduced as early as 14 days after treatment, in a dose-dependent manner. In the second case, the intravenous administration of the AAV8-desmhSGCG product in the g-sarcoglycan null mouse by the systemic route is safe and efficient. In addition to defining the dose in the resting state, there was efficiency in gene transfer in the condition of muscular mechanical challenge associated with physical effort. Thus, in resting mice, the trend toward normalization of circulating biomarkers (miRNAs + CK) correlated with the viral dose, while in mice subjected to stress, mechanical normalization was obtained only at the highest viral dose. At lower and intermediate doses, these biomarkers showed similar or even increased levels in mechanically stressed mice compared to untreated control mice.

Collectively, this analysis of the response in the treated mice confirms that miRNAs are useful as therapeutic monitoring biomarkers in LGMD.

Compared to studies performed on patients, Matsuzaka et al. (2014) found associations of miR-1 serum levels with LGMD (upregulation), although all diseases examined in this study exhibited no statistically significant associations with these miRNA levels in serum by Bonferroni’s correction. This fact may have occurred due to the small number of participants and the grouping of different LGMD, without a specific diagnosis, which may have different pathogenic mechanisms. In muscle, the answer appears to be reversed (Matsuzaka et al., 2014). In the study by Rosales and Aguennouz, a reduced expression of miR 1 was seen, which worsens with the progression of muscle damage (LGMDR1) and in groups of secondary calpain deficiency (LGMDR1 and R2), respectively, which can help in the differential diagnosis, progression assessment, and in therapeutic studies (Rosales et al., 2013; Aguennouz et al., 2016).

MiR-133 follows the pattern of miR-1: no difference in the first study; fiber downregulation with greater disease progression in the second study; and downregulation in all groups in the third study. The miR-206 showed a different response from the two previous ones, tending to up-regulate in the muscle, which may be associated with severity and progression in the two studies. In the studies of the Eisenberg and Aguennouz groups, several other miRs were found to be dysregulated (Eisenberg et al., 2007; Aguennouz et al., 2016).

Regarding the differential expression of microRNA in serum and muscle, in general, there was an inverse regulation in serum and muscle for miR-1 and -206 (the latter variable), which is upregulated in serum, and downregulated in muscle. For miR-133, there was no differential expression in serum and for the other miRs, the analysis was performed only in muscle.

Several studies have identified miR-1, miR-133a, and miR-206 as being highly enriched in the serum of mdx mice and DMD patients relative to controls (Cacchiarelli et al., 2011; Mizuno et al., 2011; Roberts et al., 2012) and animal models of LGMD (Israeli et al., 2016, 2019). Despite the significant upregulation of these miRNAs in serum, this pattern of expression is not reciprocated in skeletal muscles (i.e., miR-206 is only increased ∼4–10-fold, and miR-1 and miR-133a are generally decreased or not significantly changed in the skeletal muscles, but all three miRNAs are ∼50-fold increased in serum). Similarly, the patterns of expression of other dystromirs did not generally correlate between the muscles and serum. Thus, extracellular miRNAs do not simply result from damaged muscle due to impaired sarcolemmal integrity, and the extracellular miRNAs may constitute a specific biological response. The miR-1, miR-133a, and miR-206 can be actively released from dystrophic muscle and are protected from RNase-mediated degradation by either encapsulation in microvesicles (Hunter et al., 2008) or complex with proteins such as Argonate2 (Arroyo et al., 2011). It is speculated that these extracellular miRNAs function to drive muscle regeneration in response to muscle damage or the dystrophic condition through cell-to-cell communication.

## 5 Conclusion

The selected experimental studies addressed the relationship between miRs and the severity, progression, and/or therapeutic response of animal models of LGMD (all mice) to explore the pathophysiological aspects of this condition, using miRs as a link that could corroborate the results found.

The joint results point to the existence of a correlation between the biomarkers used and the severity and progression of LGMD animal models, as well as the alteration in the expression of miRNAs in response to gene therapy. There was, above all, but not limited to, analysis of dystromyomicroRNAs in the topics covered.

Although it is not fully understood how all epigenetic processes occur (and therefore should continue to be explored), there is sufficient evidence for the use of miRs in clinical research, and due to the complexity of interpreting the findings, preference should be given to the miRs described so far.

## Author contributions

MO, TS, WL, and LC: conception and design. MO, TS, and LC: collected the data. MO, TS, FM, JS-F, MC, GB, and LC: analysis tools. MO, TS, FM, WL, LC: performed the analysis assay. MO, WL, and LC: writing the manuscript. MO, FM, JS-F, MC, GB, WL, and LC: critical revision of the manuscript. WL and LC: obtained funding. LC: overall responsibility. All authors have read and agreed to the published version of the manuscript.
